# Editorial: Evidence-based approaches in aging and public health

**DOI:** 10.3389/fpubh.2024.1391432

**Published:** 2024-04-04

**Authors:** Brijesh Sathian, Edwin van Teijlingen, Padam Simkhada, Russell Kabir, Hanadi Al Hamad

**Affiliations:** ^1^Geriatrics and Long Term Care Department, Rumailah Hospital, Hamad Medical Corporation, Doha, Qatar; ^2^Faculty of Health and Social Sciences, Bournemouth University, Bournemouth, United Kingdom; ^3^School of Human and Health Sciences, University of Huddersfield, Huddersfield, United Kingdom; ^4^School of Allied Health, Anglia Ruskin University, Essex, United Kingdom

**Keywords:** aging research, statistical modeling, clinical trials, evidence-based medicine, collaboration

## Introduction

The past decade has seen a significant increase in aging research, driven by scientific breakthroughs and the need created by a growing global aging population. Clinical trials have made remarkable progress in providing insights into the underlying mechanisms of aging and exploring new treatments. However, it is crucial to recognize that human aging is a complex phenomenon that involves an intricate interplay of biological, social, cultural, and environmental factors. Any effective approach to aging research must therefore embrace this complexity.

Evidence-based medicine, which relies on randomized controlled trials (RCTs) for better diagnosis and treatment, often excludes older populations due to their co-morbidities and lower adherence rates. As a result, there has been a growing interest in observational studies based on real-world settings and data. These studies can supplement RCTs, monitor long-term cost-effectiveness and safety, and develop guidelines for treatment use. Given the significant impact of aging on health, finance, the economy, and society, an evidence-based approach is crucial for understanding disease causes and prevention. This Research Topic “*Evidence-based approaches in aging and public health”* welcomed opinions, reviews, systematic reviews, and original research articles on essential tools, epidemiological studies, artificial intelligence (AI), and geriatric syndromes.

For many years, aging research has been conducted in isolation, with researchers focusing on specific pathways or diseases. While this approach yielded valuable insights, it often failed to consider the interconnectedness of the various factors that contribute to aging. Today, there is a growing recognition of the need for a more holistic approach to aging research. Researchers are increasingly acknowledging the importance of genetics, lifestyle, environment, and emotional well-being in shaping the aging process. This requires a shift toward more innovative research methodologies that can integrate diverse disciplines and move beyond reductionist approaches.

This change presents considerable challenges. Traditional clinical trial designs, developed for single-target interventions, may not effectively capture the full impact of interventions aimed at the intricate web of aging. Ethical considerations become paramount, necessitating nuanced informed consent procedures that account for the long-term nature of interventions and the potential for unforeseen consequences. Furthermore, fostering interdisciplinary collaboration, while enriching, requires constructing bridges between diverse scientific cultures and methodologies. “A Call to Action for Collective Efforts: The apex research institutions,” as a platform for pioneering medical advancements, must champion this critical transition. We urge scientists, clinicians, policymakers, and funding agencies to unite in supporting and nurturing to developing innovative clinical trial designs. Trials incorporating multidimensional endpoints, longitudinal assessments, and adaptive methodologies will be vital for evaluating holistic interventions.

Precision medicine has transformed cancer treatment by leveraging next-generation sequencing and tailored therapies. This has resulted in the emergence of novel trial designs, including basket and umbrella trials, master platform trials, and N-of-1 patient-centric studies. Additionally, real-world data, digital applications, and machine learning are being employed to expedite knowledge acquisition. Clinical trials have shifted from focusing on specific tumor types to being gene-directed and histology-agnostic, with personalized treatment plans tailored to individual biomarker profiles (1). Fostering interdisciplinary collaboration: encouraging partnerships between biologists, gerontologists, social and behavioral scientists, data scientists, and ethicists will be crucial for designing and implementing comprehensive aging research programs (2). Promoting public engagement and openly discussing the complexities and uncertainties of aging research, while emphasizing its potential benefits, will gain public trust and foster broader understanding.

Late-life learning programs can help answer research questions and enhance our comprehension of active and healthy aging. Such programs can be utilized to target age-related diseases such as dementia and cognitive decline. Research on the multidimensional health impact of late-life learning can strengthen national strategies and inform policies. These programs are cost-effective, scalable, and suitable for use in low-resource settings. More implementation research is needed to ensure that these programs reach vulnerable groups and older adults. The COVID-19 pandemic has accelerated the shift to online learning, promoting digital inclusion (3).

Scientific research has advanced significantly in domains like immunology and genetics over the last 30 years. Unfortunately, due to the high failure rate and inefficiencies in the healthcare system, clinical research is still moving slowly. Innovative approaches are required to solve this, in order to involve patients and produce evidence for novel medical advancements. The COVID-19 pandemic revealed structural flaws in the way clinical trials are conducted, which spurred scientists to create patient-centric trials of the future. Deep neural networks, multimodal biomedical AI, and machine learning have the potential to revitalize clinical research by enhancing image interpretation, workflow, and drug discovery (4). The fast advancements in precision medicine, immunology, and genomics require adjustments to clinical trial design. Although RCTs are regarded as the gold standard in drug discovery, they come with a high price and risk. Numerous practical uses of AI are being investigated as a means of achieving sustainable and optimal medication development. Utilizing AI models, data are transformed into meaningful insights, accelerating the process of drug research. Opportunities include how AI can help find focused therapeutics and rare illness treatments, improve patient recruitment and protocol design efficiency, and use AI to monitor patients. Furthermore, to help businesses decide whether to engage in AI integration and to ascertain the areas where regulation will have the greatest influence, this research attempts to identify opportunities, obstacles, and future implications for AI in RCTs (5–9).

ASReview is an open-source program that researchers have created to make the process of screening abstracts and titles for systematic reviews and meta-analyses more efficient. Only a small percentage of the screened research are deemed significant in the scientific literature, a problem that the new technology is intended to remedy. With the help of this tool, review procedures will be able to become more transparent and efficient by mitigating the error-prone and inefficient process of manually screening thousands of research publications (10).

## Contribution to the field

It is vital to recognize and take lessons from the health system measures that can improve healthy aging. Considering the need for more and better evidence-based policies and care for our aging populations, we present 15 papers from across the globe in this Research Topic on “*Evidence-based approaches in aging and public health”* of *Frontiers in Public Health*. These papers cover different research methods ranging from hospital-based study (Chen S. et al.) to community based study (Peng et al.) to epidemiological study (Jiao et al.), and from RCTs (Xu et al.), to studies based on statistical modeling (Elamin and Ansah; Ye et al.) to a review (Chen J. et al.) as well as several systematic reviews with meta-analyses (Gao et al.; Dai et al.; Li et al.; Zhou et al.)

As is to be expected from Public Health as a broad discipline, the diseases and issues addressed in the 15 papers are also wide-ranging. “*Evidence-based approaches in aging and public health*” covers diverse topics such as dentistry, sarcopenia, physical activity, sleep, Alzheimer's and Parkinson's, arterial fibrillation, hip fracture, rehabilitation, unmet needs, medical costs, reducing blood pressure, inappropriate medications and much more. Interestingly, the overwhelming majority (11/15) of papers in this Research Topic were submitted from authors based in China, with one study each focusing on Italy (De Cola et al.), the United Kingdom (Elamin and Ansah), Singapore (Ansah et al.), and Vietnam (Phi et al.).

According to the Chen S. et al. study, hospitalization patterns among patients with Alzheimer's disease (AD) and Parkinson's disease (PD) varied markedly. For hospitalized patients with AD and PD, it is crucial to apply alternative management, and distinct priorities should be set when developing primary preventive programs, identifying care needs, and allocating healthcare resources.

Peng et al. investigated cognition and physical frailty in older persons and found that sleep quality partially mediates the association between cognitive impairment and physical frailty.

Jiao et al. reported that there are notable regional and national differences in the illness burden of atrial fibrillation (AF). In terms of incident cases [818,493 (562,871–1,128,695)], deaths [39,970 (33,722–46,387)], and disability-adjusted life years (DALYs) [1,383,674 (1,047,540–1,802,516)] at the national level, China topped among the list of nations.

Patients with mild cognitive impairment (MCI) benefit significantly from Tai Chi in addition to transcranial direct current stimulation (tDCS) for improved global cognitive performance, memory, execution function, and attention (Xu et al.). These results point to the possible application of Tai Chi and tDCS as a non-invasive brain stimulation regimen and physical exercise to enhance cognitive function in older persons with MCI.

Using a multi-state population model, Elamin and Ansah estimated the prevalence of periodontal diseases and dental caries in the adult population in the United Kingdom. This model gives policymakers a realistic, evidence-based estimate of future demand for oral health issues. Due to the considerable time lag in the education and training of oral health professionals, these forecasts allow policymakers to anticipate future capacity demands proactively rather than reactively.

The nature of sarcopenia transitions and estimations of life expectancy with and without sarcopenia are addressed by Ye et al.. Their results underscore the significance of early detection and treatment for sarcopenia among older Chinese people, enhancing our knowledge of the connection between sarcopenia and life expectancy, and offering targeted health education. They noted that sarcopenia is more common in women, older adults with low education, single people, those with an agriculture hukou, and smokers, both current and past. Targeted interventions are needed to increase the number of older people in western China's impoverished and rural areas who do not have sarcopenia.

Chen J. et al. reviewed factors that influence older persons' medical expenses. The medical costs of older persons need to be evaluated more thoroughly since they are more likely to develop chronic illnesses. Financing strategies, multidimensional comparisons, and factor investigations should all be used to analyse the medical costs incurred by older persons. Furthermore, research should be done on how rising medical expenses affect Medicare 'financing for older adults, healthcare services, and medical insurance support programs. In order to lessen the burden on older persons, policy makers should focus more on the medical costs of these individuals and the factors that influence them and develop relevant policies in a multifaceted and all-encompassing manner.

According to a network meta-analysis by Gao et al., middle-aged and older persons can significantly lower their blood pressure with both static and aerobic exercise. Both exercise modalities had a considerable impact on systolic blood pressure, but not diastolic blood pressure. The SUCRA ranking results indicate that for middle-aged and older persons, static exercise lowers blood pressure more effectively than aerobic exercise, and for those with hypertension, appropriate exercise can lower blood pressure to some extent.

In a meta-analysis by Dai et al., providing traditional Chinese exercises (TCEs) to patients with chronic heart failure (CHF) had a positive impact on their recovery, primarily by improving LVEF, VO_2_max, anaerobic threshold, quality of life, and single-item traditional Chinese medicine scores (fatigue, palpitations, floating limbs, and shortness of breath).

According to Li et al. meta-analysis, lifestyle modification (LSM) is advised as a long-term BP (blod pressure) control regimen; additionally, TCD bubble is suggested for lowering SBP and RE as a potential means of lowering BP. Aerobic exercise on its own or combined with resistance exercise and dietary approaches to stop hypertension (DASH) are recommended for the Prehypertension (PHT) population with moderate to high quality evidence for BP lowering.

According to Zhou et al., pharmacological interventions can minimize the incidence of potentially inappropriate medications (PIMs), the number of PIMs per person, the amount of pharmaceuticals used, and the 30-day readmission rate, all of which can enhance the prognosis of older patients.

De Cola et al. claim that the establishment of a hub-and-spoke network for intense neuro-rehabilitation has improved regional care services for neuro-rehabilitation while also facilitating the management of neurological patients by preventing needless long-distance travel.

To promote system-wide solutions, Ansah et al.'s research discusses the elements that either facilitate or impede hip fracture recovery. They do this by adopting a feedback perspective. Their study report that recovering from a hip fracture-related loss of function is largely dependent on two factors: (a) identifying the difference between one's pre-fracture and current physical functions; and (b) using psychological resilience to act quickly to address a functional loss through rehabilitation services.

According to the results of the (Phi et al.) study, a large number of older Vietnamese individuals had functional impairments. Those not marital (divorced, separated, single) had the highest percentage of unmet demands but the lowest rate of care needs among this group. Unmet needs were more common among rural people with poorer health than among those who lived in cities and had normal or fair health.

## Conclusion

The past decade has seen increased research on aging, driven by scientific breakthroughs and a growing aging population. Clinical trials have made progress in targeting aging mechanisms, but a more holistic approach is needed. There is a growing interest in observational studies and real-world data. This editorial recognizes the need to adopt “Complexity in Aging Research,” and hence we call for more interdisciplinary collaboration and public engagement. A shift toward more innovative research methodologies that integrate diverse disciplines is required. This change presents significant challenges, such as ethical considerations and interdisciplinary collaboration. The research institutions must champion this critical transition by supporting innovative clinical trial designs and fostering interdisciplinary collaboration. Encouraging partnerships between biologists, gerontologists, social scientists, data scientists, and ethicists will be crucial for designing and implementing comprehensive aging research programs. Late-life learning programs can help target age-related diseases such as dementia and cognitive decline. These programs are cost-effective, scalable, and suitable for use in low-resource settings.

## Author contributions

BS: Conceptualization, Data curation, Methodology, Project administration, Supervision, Validation, Writing – original draft, Writing – review & editing. EvT: Conceptualization, Data curation, Methodology, Supervision, Writing – original draft, Writing – review & editing. PS: Methodology, Validation, Writing – review & editing. RK: Methodology, Validation, Writing – review & editing. HA: Methodology, Supervision, Validation, Writing – review & editing.
